# Research progress on the analysis of resistance genes and mechanisms of wheat fusarium crown rot

**DOI:** 10.3389/fpls.2025.1603842

**Published:** 2025-10-07

**Authors:** Heng Zhou, Wenxin Cao, Yao Li, Qiqi Zhang, Fangfang Liu, Yingxiu Wan

**Affiliations:** Crop Research Institute, Anhui Academy of Agricultural Sciences/Anhui Key Laboratory of Crop Quality Improvement, Hefei, Anhui, China

**Keywords:** fusarium crown rot, gene discovery technology, disease-resistant gene, disease resistance mechanism, signal transduction

## Abstract

Fusarium crown rot (FCR) of wheat represents a critical challenge to global wheat production. Discovering disease-resistant genes and analyzing their resistance mechanisms are crucial for breeding resistant varieties and controlling the disease. In recent years, molecular biology and genomics technologies have advanced rapidly. This has enabled remarkable progress in discovering FCR-resistant genes in wheat. Through genetic mapping, association analysis, and mutant screening, multiple gene loci related to wheat FCR resistance have been identified. For instance, the gene locus Qfcr.sicau-4B on chromosome 4B was found to significantly enhance FCR resistance by regulating cell wall lignification, while the Fhb1 locus on chromosome 3B, though originally identified for fusarium head blight resistance, has shown cross-resistance to crown rot in some genetic backgrounds. In terms of mechanism analysis, studies show that these resistant genes combat pathogen invasion through multiple pathways. For example, they can activate the plant immune system, regulate defense-related gene expression, enhance cell wall structural stability, and mediate reactive oxygen species (ROS) metabolism. The ROS detoxification pathway, exemplified by the *TaCAT1* gene encoding catalase, efficiently scavenges hydrogen peroxide to prevent oxidative damage during pathogen infection. Additionally, the mitogen-activated protein kinase (MAPK) cascade pathway, such as the TaMPK3-TaMPK6 module, has been shown to phosphorylate and activate transcription factors that induce defense gene expression. Additionally, signal transduction pathways play a bridging role in resistant gene function. Pathways such as the MAPK cascade and plant hormone signal transduction are involved in transmitting and amplifying resistance signals. This review systematically reviews methods for discovering wheat FCR-resistant genes, identified resistant genes and their functions, and deeply analyzes resistance mechanisms. Its aim is to provide a theoretical basis and technical support for genetic improvement and sustainable control of wheat FCR.

## 1 Introduction

**Figure 1:**
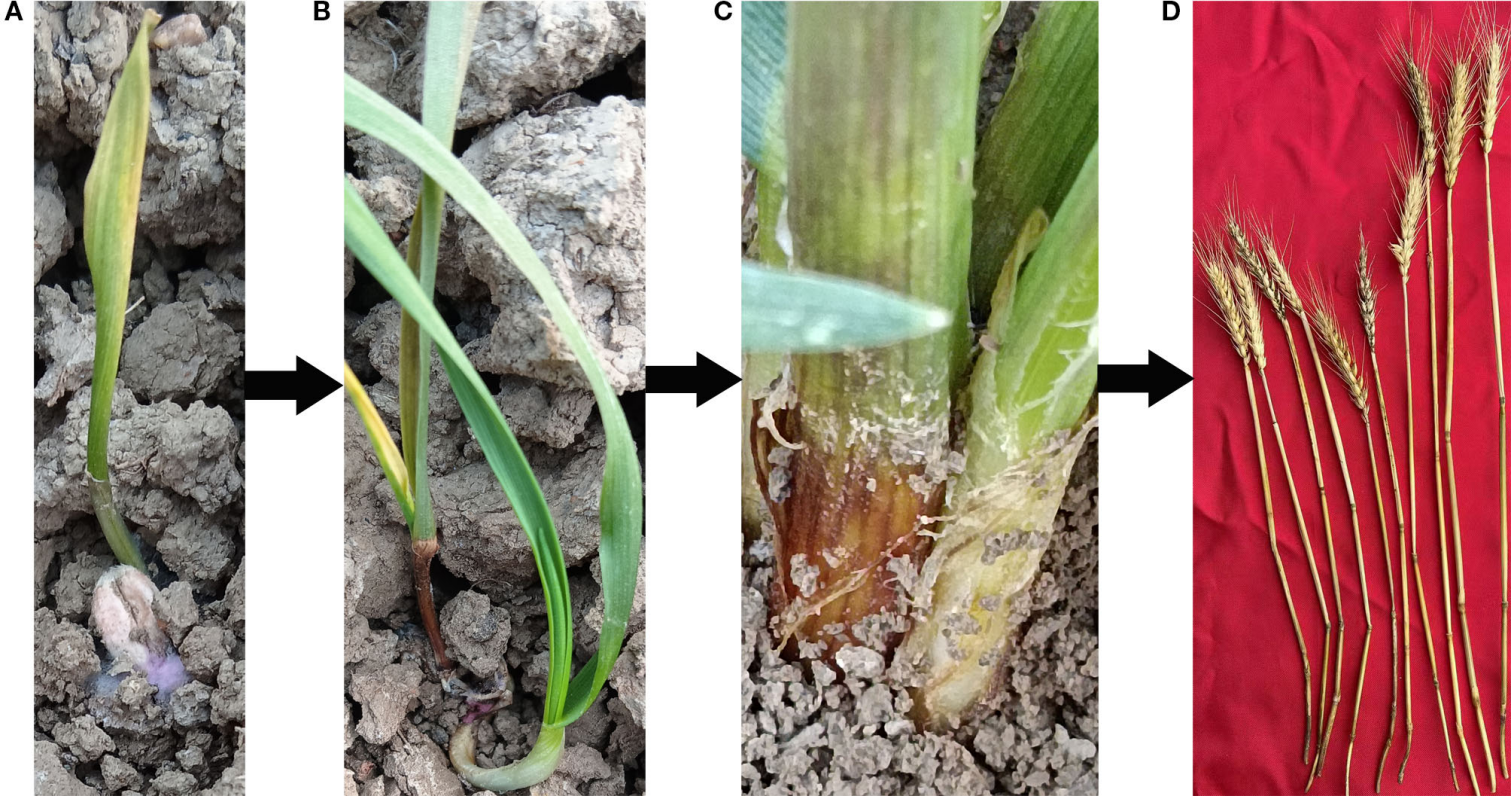
The pathogenesis process of wheat after infection by stem base rot pathogen. **(A)** Wheat grain disease occurrence; **(B)** Wheat seedling stage stem base disease occurrence; **(C)** Wheat adult plant stage disease occurrence; **(D)** Wheat mature stage disease occurrence.

Fusarium crown rot (FCR) of wheat is a global soil-borne disease, which was first reported in Australia in 1940 (McKnight and Hart, 1966), and has now spread into a worldwide disease (Shi et al., 2024). In the Huang-Huai wheat region of China, FCR occurs commonly and causes serious damage in Henan Province (Xu et al., 2025). It is also severe in major wheat-producing areas such as Shandong and Anhui provinces, having a great impact on the yield and quality of wheat. In severely affected fields, the yield loss can be as high as 38% - 61% (Xu et al., 2016; Wu et al., 2018; Lin et al., 2023; Fan et al., 2021). The main pathogenic bacteria of wheat FCR include Fusarium pseudograminearum, Fusarium culmorum, Fusarium graminearum, etc (Smiley and Machado, 2020). Currently, the vast majority of wheat varieties identified at home and abroad are susceptible to FCR, and the disease can occur throughout the entire growth and development process of wheat. During the seed germination stage, the pathogen will inhibit the elongation rate of the coleoptile and the emergence rate. An excessive amount of the pathogen will cause the seeds to rot or the seedlings to wither, leading to the lodging or death of the seedlings. In the seedling stage, the base of the infected plant’s stem begins to turn brown. In severe cases, the plant’s stem turns brown and rots, and may even lodge and die. In the middle and later stages, the disease spreads to the wheat stem, and the internodes are prone to breakage, showing brown necrosis, and pink mycelium can be seen around the stem. In the later stage, it can cause white ears in wheat, resulting in empty grains or no seeds, leading to yield reduction(**Figure 1**) (Brennan et al., 2003; Kettle et al., 2015; Glenn et al., 2002; Huo et al., 2010; Mitter et al., 2006; Smiley et al., 2005). During the infection process of FCR, various toxins such as deoxynivalenol (DON) are also produced, which seriously endanger the health of humans and livestock (Monds et al., 2005; Beccari et al., 2018). Wheat FCR caused by Fusarium pseudograminearum was first reported in Qinyang, Henan Province, China in 2012 (Xu et al., 2025). Due to the general susceptibility of wheat varieties in production, and the large-scale promotion of measures such as returning straw to the field in recent years, the disease continues to spread and intensify in China. In June 2022, the question of “Why has wheat Fusarium crown rot (FCR) broken out in major wheat-producing areas of China in recent years, and how can it be scientifically and effectively prevented and controlled?” was listed as one of the top 10 industrial technology-related issues associated with industrial development by the China Association for Science and Technology. Since the pathogen can survive on the diseased residues in the soil for a long time and spreads rapidly to cause disasters when the climate is suitable, it is difficult to control. In the face of this disease threat, traditional chemical control methods are not only costly but also prone to problems such as environmental pollution and the enhancement of pathogen resistance, making it difficult to achieve sustainable control. Cultivating and planting disease-resistant varieties is recognized as the most economical, effective, and environmentally friendly strategy for preventing and controlling wheat FCR. Discovering disease-resistant genes for wheat FCR and deeply analyzing their disease resistance mechanisms are the core and prerequisite for breeding disease-resistant varieties. By clarifying the genetic basis and molecular mechanisms of wheat resistance to FCR, it can provide accurate theoretical guidance and gene resources for disease-resistant breeding, and fundamentally improve the resistance of wheat to FCR. In recent years, with the rapid development of technologies in the field of life sciences, especially the extensive application of technologies such as molecular biology and genomics, it has provided powerful tools and means for the discovery of disease-resistant genes for wheat FCR and the analysis of their mechanisms, enabling a series of important progress to be made in this field. This review will systematically review these achievements, aiming to comprehensively sort out the methods for discovering disease-resistant genes for wheat FCR, the identified disease-resistant genes and their functions, and deeply analyze the current research status of disease resistance mechanisms, in order to provide useful references for further research on the prevention and control of wheat FCR and the breeding of disease-resistant varieties, and to promote the healthy and sustainable development of the wheat industry.

## 2 Evolution of research on wheat fusarium crown rot

**Figure 2:**
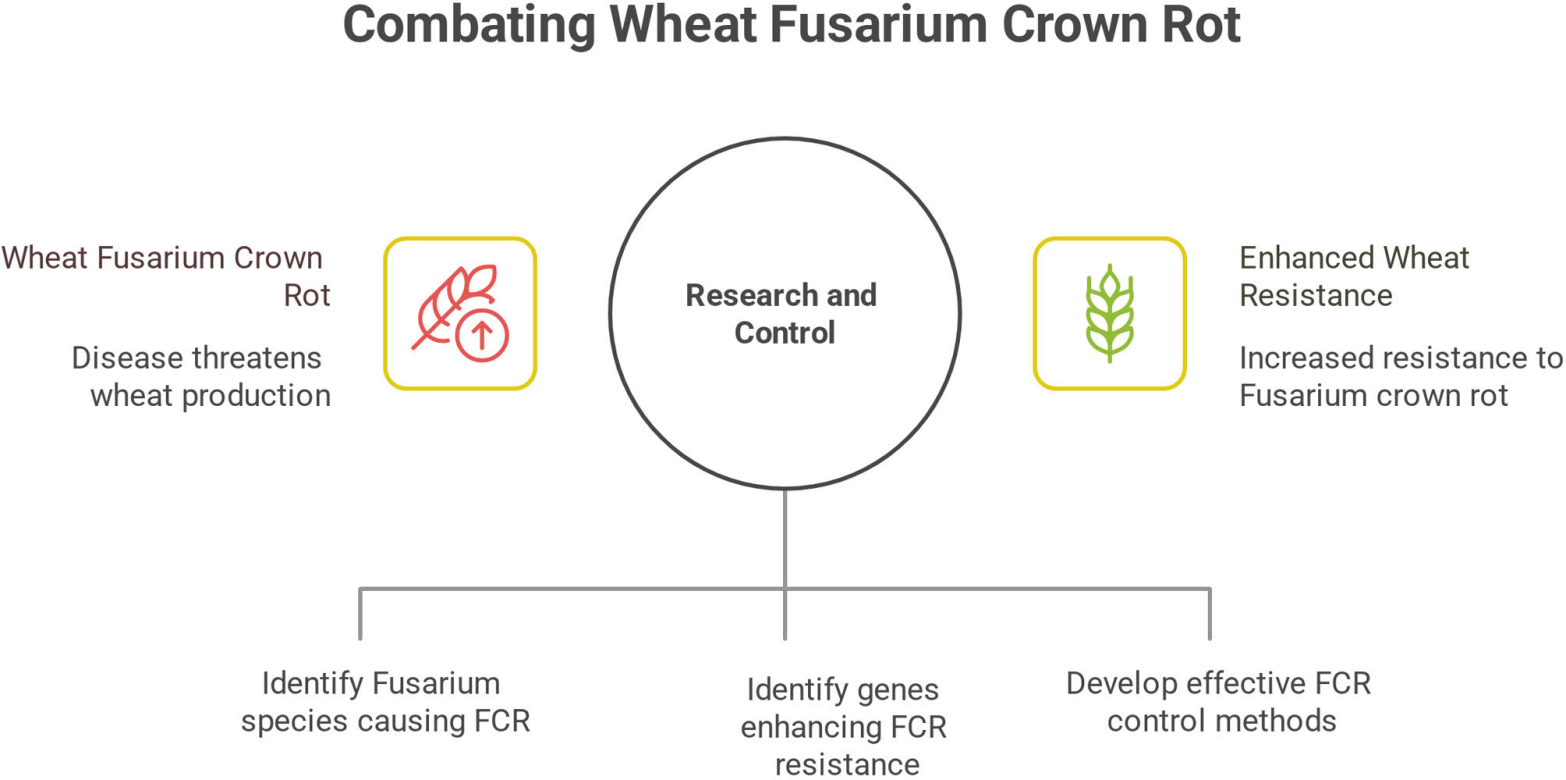
Evolution of research on wheat fusarium crown rot. 1950–2012: Fusarium crown rot (FCR) of wheat was first reported internationally. During this period, the primary causal pathogens were identified, and variations in cultivar resistance were documented. 2012–2015: In China, FCR caused by Fusarium pseudograminearum was first identified and reported. Research efforts focused on pathogen identification, distribution mapping, and damage assessment. 2015–2022: It was revealed that FCR in China is caused by multiple Fusarium species, with the dominant pathogens varying across different regions. Additionally, the key drivers underlying disease spread were clarified. 2022–2025: Studies have centered on the mining of disease resistance genes and pathogenicity genes, along with the elucidation of resistance and pathogenic mechanisms.

1950–2012: Initial Discovery and Observation. Wheat Fusarium crown rot was first reported in Australia in the 1950s, with early studies primarily focusing on preliminary observation and documentation of the disease, confirming its existence as a novel wheat pathogen (Li et al., 2024a). During this period, initial research in China also emerged. For example, Tian et al. (2015) isolated seven fungal species from diseased wheat stem bases, identified Rhizoctonia cerealis and Fusarium nivale as the main pathogens, and observed significant resistance variations among wheat cultivars. 2012–2015: Onset of Systematic Research in China. In 2012, wheat FCR caused by Fusarium pseudograminearum was first identified and reported in China, prompting increased domestic research on the disease (Li et al., 2024a). This stage focused on pathogen identification, distribution mapping, and damage assessment, laying the foundation for subsequent in-depth studies. 2015–2022: Mechanistic Insights and Epidemiology. Since 2015, systematic research on wheat FCR has been conducted (Li et al., 2024a). Through systematic sampling, morphological and molecular identification, it was revealed that FCR in China is caused by multiple Fusarium species, with dominant pathogens varying by region. Key drivers of disease expansion were identified, including poor cultivar resistance, pathogen accumulation in soil due to long-term straw return, and deteriorated soil ecological conditions (Li et al., 2024a). 2022–2025: Breakthroughs in Resistance and Control. In 2022, the Chinese Association for Science and Technology listed wheat FCR as one of the top ten industrial technical issues in China, highlighting its critical impact on wheat production and accelerating research (Sun et al., 2024). In 2023, the wheat receptor-like kinase gene TaRLK-6A was identified, which positively regulates defense gene expression to enhance FCR resistance, providing a candidate gene for molecular breeding (Qi et al., 2024). The succinate dehydrogenase inhibitor (SDHI) cyclobutrifluram showed effective control of FCR, though moderate resistance risk in pathogens was confirmed (Sun et al., 2024). Population genomic analysis revealed that geographical distribution differences between 3AcDON and 15AcDON strains of F. pseudograminearum are closely associated with secondary metabolite synthesis genes (Sun et al., 2024). The Zn2Cys6 transcription factor gene Fp487 was found to play critical roles in F. pseudograminearum development and virulence, emerging as a potential RNAi-based control target for FCR (Sun et al., 2024). In 2025, the FCR resistance gene TaCAT2 was cloned, unveiling a novel resistance mechanism mediated by TaCAT2(**Figure 2**) (Yang et al., 2025).

## 3 Population diversity of pathogens causing wheat fusarium crown rot

Wheat Fusarium crown rot (FCR) is caused by complex infections of multiple pathogens, primarily including: *Fusarium graminearum*: A dominant pathogen worldwide, causing both Fusarium head blight (FHB) and FCR. It produces deoxynivalenol (DON), a mycotoxin threatening food safety. *Fusarium avenaceum*: Widespread in temperate humid regions (Europe, North America), tolerant to low temperatures, and forming competitive or synergistic infections with *Fusarium graminearum*. *Fusarium culmorum*: Common in European and North China wheat regions, preferring sandy loam soils, and causing brown necrosis at the stem base. Other pathogens: *Fusarium pseudograminearum* (predominant in Australian wheat regions), *Fusarium* sp*orotrichioides* (producing T-2 toxin). (**Table 1**) (Qi et al., 2024). In China’s Huang-Huai-Hai wheat region, *F. graminearum* and *F. culmorum* are dominant. In the Yangtze River basin, *F. avenaceum* prevails due to high humidity. The North American Great Plains are dominated by *F. graminearum*, while Nordic wheat regions exhibit mixed infections of *F. culmorum* and *F. avenaceum*. Single resistance genes (the major FHB QTL Fhb1) are prone to losing efficacy against pathogen population variations due to the evolution of virulence genes (Yang et al., 2025). Single resistance genes are easily overcome by new pathogen virulent races. Pyramiding 2–3 major genes or combining multiple minor-effect QTLs is essential. Establish a national pathogen virulence monitoring network to regularly assess resistance gene effectiveness. For example, track virulence gene evolution via whole-genome sequencing. Use biocontrol agents (*Trichoderma harzianum*) or organic amendments to modulate rhizosphere microbial communities, reducing pathogen virulence and delaying resistance gene decay. Elucidate the molecular mechanisms of pathogen-wheat interactions, such as recognition patterns between effectors and resistance proteins. Develop a “resistance gene matching model” based on pathogen population genomics to predict optimal breeding combinations and enhance the foresight of resistance deployment.

**Table 1:** Population diversity of pathogens causing wheat fusarium crown rot.

Fusarium species	Distribution and characteristics
*Fusarium graminearum*	Global pathogens can simultaneously cause Fusarium head blight and Fusarium crown rot; they produce deoxynivalenol.
*Fusarium avenaceum*	Widely distributed in temperate humid regions (such as Europe, North America), with low-temperature resistance; often forms competitive or synergistic infections with *Fusarium* graminearum.
*Fusarium culmorum*	Common in wheat-growing areas of Europe and northern China, preferring sandy loam soil environments; infection leads to brown necrotic symptoms at the base of wheat stems.
Other pathogens	*Fusarium pseudograminearum*: Dominant in wheat-growing areas of Australia. *Fusarium* sp*orotrichioides*: Produces T-2 toxin.

## 4 Gene discovery methods and technologies

### 4.1 Traditional genetic mapping methods

Traditional genetic mapping methods mainly rely on constructing genetic maps based on biparental populations, and then conducting quantitative trait locus (QTL) mapping (Zhao et al., 2019; Zhang et al., 2021). The principle is to use the linkage relationship between molecular markers and the genes of the target trait. By analyzing the segregation of markers and traits in the offspring population, the position of the gene on the chromosome is determined (Cheng, 2013; Chimthai et al., 2025; Gu et al., 2024; Ye, 2009; Darvasi et al., 1993). Traditional genetic mapping methods have played an important role in the discovery of genes resistant to wheat FCR and laid the foundation for subsequent research. However, this method also has certain limitations, such as the need to construct a special genetic population, which takes a long time, has relatively low positioning accuracy, and is easily affected by environmental factors.

### 4.2 Genome-wide association study

Genome-wide association study (GWAS) is a gene-mapping approach based on natural populations, which leverages the rich genetic variations within these populations to identify trait-associated loci by detecting the association between numerous molecular markers (e.g., single-nucleotide polymorphisms, SNPs) and target traits (Morris et al., 2013). In wheat Fusarium crown rot (FCR) research, the application principle of GWAS is as follows: First, natural wheat populations with broad genetic diversity are collected, including different cultivars, lines, and local germplasms (Li et al., 2024a). For instance, recent GWAS populations commonly include typical cultivars such as Chinese Spring, Yangmai 18, and Bobwhite: Chinese Spring serves as a model cultivar providing a reference framework for whole-genome sequencing; Yangmai 18, as a major cultivar in southern China, is used for resistance phenotype association analysis; and Bobwhite has become an international standard material for disease resistance gene mapping due to its clear genetic background (Li et al., 2024a; Wang et al., 2024; Yu, 2021). GWAS offers numerous advantages: it eliminates the need to construct special genetic populations, directly utilizing existing natural populations to significantly shorten the research cycle; it enables simultaneous detection of multiple loci across the whole genome, rapidly locating target trait-related gene regions and improving gene discovery efficiency; and due to the rich genetic diversity of natural populations, GWAS can detect more genetic variations and identify minor-effect genes that are difficult to detect by traditional methods. However, GWAS also has certain limitations: it requires a large number of samples and high-density molecular markers, involving high costs; results are susceptible to population structure and linkage disequilibrium, potentially leading to false-positive or false-negative outcomes; and GWAS can only determine the association between markers and traits, making it difficult to directly identify causal genes, which requires further verification and functional analysis.

### 4.3 Transcriptomic analysis

Transcriptomic analysis is a discipline that investigates the types, structures, and expression levels of all transcripts in a specific cell, tissue, or organism under a given condition. In the discovery of genes associated with wheat Fusarium crown rot (FCR), transcriptomic analysis screens for differentially expressed genes (DEGs) by comparing transcriptomic changes in wheat before and after pathogen infection, thereby identifying disease-resistance-related genes. For example, a high-throughput panoramic map of transcriptome, proteome, phosphoproteome, and acetylome across 20 tissues throughout the entire growth period of wheat was constructed. Through multi-omics analysis, the TaHDA9-TaP5CS1 module was identified. Infection by Fusarium pseudograminearum downregulated the expression of TaHDA9 in FCR-resistant wheat plants, thereby relieving the acetylation restriction imposed by TaHDA9 and increasing the expression of TaP5CS1. This led to an elevation in proline content, which in turn enhanced wheat resistance to FCR (Zhang et al., 2025).

### 4.4 Gene editing technology-assisted verification

**Figure 3:**
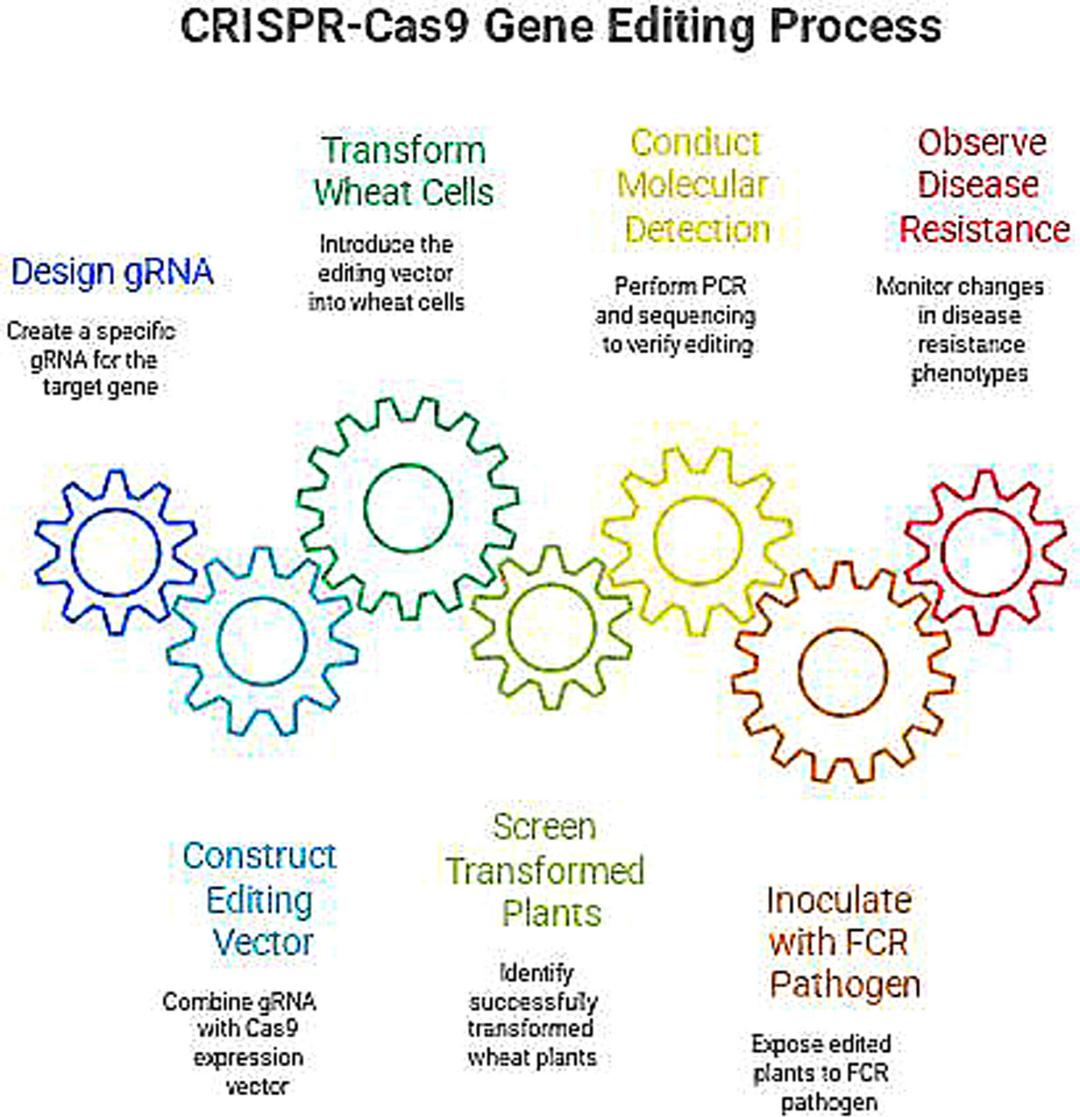
Schematic diagram of target gene knockout by the CRISPR/Cas9 system.

Gene editing technology is a technology that can precisely modify the genome of an organism. In the discovery of genes related to wheat FCR, it is mainly used to verify the function of genes. Among them, the CRISPR/Cas9 technology has become the most commonly used gene editing tool due to its advantages such as simple operation, high efficiency, and strong specificity. The CRISPR/Cas9 system consists of the Cas9 nuclease and the guide RNA (gRNA). The gRNA can recognize and bind to a specific sequence of the target gene, guiding the Cas9 nuclease to cut this sequence, generating a double-strand break (DSB). During the repair of the DSB in the cell, non-homologous end joining (NHEJ) or homologous recombination (HR) will occur, thus achieving modifications such as knockout, insertion, or replacement of the target gene. In the verification of genes related to wheat FCR, first design a specific gRNA according to the sequence of the target gene, and connect it with the Cas9 nuclease expression vector to construct a gene editing vector. Use methods such as Agrobacterium-mediated transformation and particle bombardment transformation to introduce the gene editing vector into wheat cells. Screen out the successfully transformed wheat plants, and conduct molecular detection on them, such as PCR, sequencing, etc., to determine whether the target gene has been successfully edited. Conduct an inoculation experiment of the FCR pathogen on the edited wheat plants, and observe the changes in their disease resistance phenotypes. If the resistance of the wheat plants to FCR is significantly reduced after knocking out a certain gene, it indicates that this gene may positively regulate the disease resistance of wheat; conversely, if the resistance of the wheat plants is enhanced after overexpressing a certain gene, then this gene may be a disease resistance-related gene(**Figure 3**). Silencing of TaALDHase could significantly increase wheat resistance to FCR. However, interference with TaWRKY24 or TaMTase could decrease wheat resistance to FCR (Xu et al., 2024). In addition to the CRISPR/Cas9 technology, gene editing technologies such as TALEN (Transcription Activator-Like Effector Nucleases) and ZFN (Zinc Finger Nucleases) have also played a certain role in gene function verification. These technologies provide powerful means for the functional verification of genes resistant to wheat FCR, help to deeply understand the molecular mechanisms of wheat disease resistance, and accelerate the application of disease-resistant genes.

## 5 Progress in the discovery of genes related to wheat FCR

### 5.1 Identification of germplasm resources

Planting disease-resistant varieties is the most economical and effective measure to deal with wheat FCR. Therefore, screening and identifying wheat germplasm resources resistant to FCR is of great significance (Zhang et al., 2024). For a long time, it have been committed to this work and achieved certain results in Australia. For example, disease-resistant germplasms such as 2-49, CSCR6, and Sunco have been screened out. These germplasms have shown resistance to wheat FCR in different studies, providing valuable materials for subsequent research and breeding work (Li et al., 2024a). In recent years, Chinese scholars have also increased the intensity of identifying germplasms resistant to FCR, and have successively identified the resistance of more than 1500 wheat lines (Li et al., 2024a). However, regrettably, the identification results show that the proportion of disease-resistant germplasms is less than 10%, indicating that the germplasm resources resistant to FCR in China’s wheat are relatively scarce. The main reason for this situation is that there are great differences in the resistance identification methods (Li et al., 2024a). Currently, there are various methods for identifying the resistance to wheat FCR, including indoor seedling stage identification and field adult plant stage identification (Li et al., 2008).

#### 5.1.1 Indoor seedling stage identification

**Figure 4:**
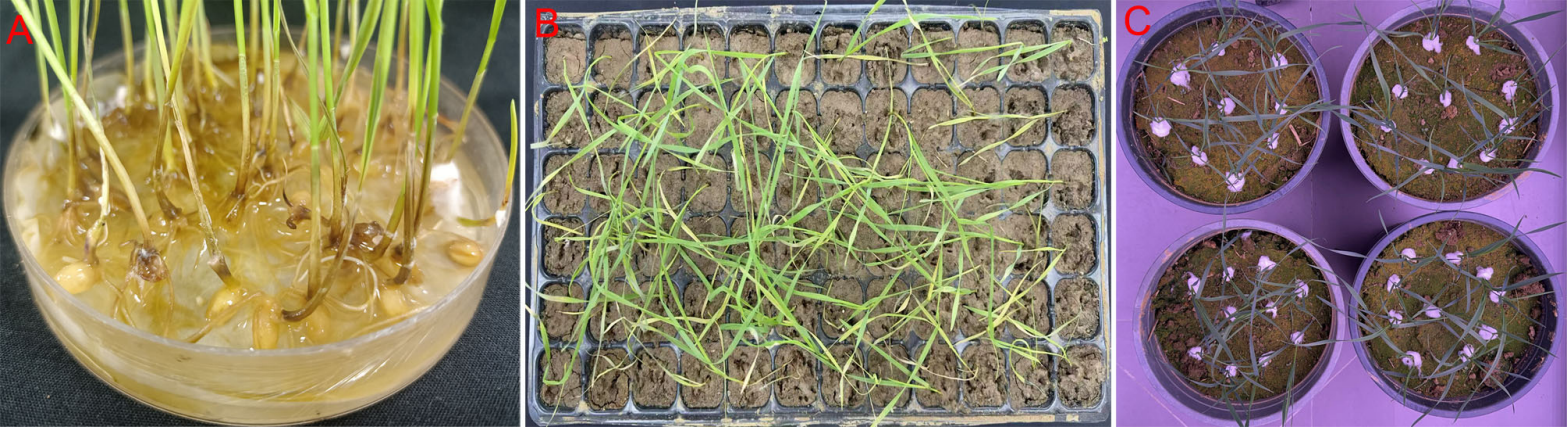
Three seedling inoculation methods. **(A)** Petri dish seedling direct inoculation culture method; **(B)** Bacterial solution seed soaking inoculation method; **(C)** Cotton ball inoculation method.

Commonly used effective methods for indoor seedling stage identification include the Petri dish seedling direct inoculation culture method, bacterial solution seed soaking inoculation method and cotton ball inoculation method(**Figure 4**) (Huo et al., 2010; Li et al., 2008, 2024a). Among the three methods, the spore suspension seed soaking method features simple operation and high inoculation efficiency, making it suitable for large-scale primary screening of germplasms. The cotton ball inoculation method allows precise control of the inoculation site and is frequently used in resistance mechanism research. The natural substrate inoculation method is closer to the field disease environment and is suitable for re-screening of resistant germplasms. It should be noted that the inoculation concentration, culture temperature, and investigation time of different methods vary significantly (for example, the concentration of the spore suspension method is at the 10^6^ level, while that of the cotton ball method is at the 10^5^ level), which may lead to inconsistent resistance performance of the same germplasm under different methods (Li et al., 2024a). Therefore, in practical applications, it is necessary to select appropriate methods according to the research purpose and standardize the operation process (**Table 2**).

**Table 2:** Identification of disease grades for stem base rot.

Disease grade	Disease symptoms
0	healthy, with no symptoms on the outer sheath
1	the lesion area of the first sheath is less than 1/4 of the length of the sheath
2	the lesion area of the first sheath is 1/4–1/2 of the length of the leaf sheath
3	the lesion area of the first sheath is 1/2–3/4 of the length of the leaf sheath, and the disease affects the inner leaf sheath
4	the first sheath is completely degreened and putrid, or the second sheath has obvious browning
5	the third leaf sheath has obvious brown blight, or the whole plant has died

#### 5.1.2 Field adult plant stage identification

Field adult plant stage identification mainly uses the natural medium method, sowing the pathogen-infected millet or diseased wheat grains in the field, mixing them with the surface soil and then sowing, or placing wheat seeds in a plastic tube covered with pathogen-infected soil, and then burying the plastic tube in the field (Li et al., 2024a). Different identification methods have differences in inoculation methods, inoculation concentrations, culture conditions, disease investigation time, and evaluation criteria, which leads to inconsistent identification results of some germplasms and unstable resistance of some disease-resistant germplasms (Li et al., 2024a). For example, some germplasms show resistance under one identification method, but show susceptibility under another method, which brings great difficulties to the screening and utilization of germplasm resources.

### 5.2 Discovery of resistance loci

The resistance to Fusarium crown rot (FCR) in wheat is a quantitative trait, which is controlled by multiple genes. The broad-sense heritability ranges from 0.19 to 0.98 (Li et al., 2024a). Identifying major quantitative trait loci (QTL) is of great significance for accelerating the process of disease-resistant breeding (Wang et al., 2024),. Early studies mainly focused on mapping QTLs related to FCR resistance based on biparental recombinant inbred line (RIL) or double haploid (DH) populations (Li et al., 2024a). A large number of stem base rot resistance loci have been identified (Hou et al., 2023; Martin et al., 2015; Jin et al., 2020; Bovill et al., 2010; Yang et al., 2019; Poole et al., 2012)(**Table 3**).These studies have laid the foundation for the discovery of resistance loci to FCR in wheat.

**Table 3:** Research progress of genome-wide association study.

Gene name	Chromosome	Linked marker	Function	Physical position	References
Qfcr. sicau. 1A-1	1A	wsnp_Ku_c183_358844	Resistance to FCR	8. 33 ~ 14. 33	Hou et al. (2023)
Qcr-Xbarc148	1A	barc148-gwm164	Resistance to FCR	52. 22	Martin et al (2015)
Qr-Affx-109251450	1B	Affx-109251450	Resistance to FCR	56. 88	Jin et al. (2020)
Qcr. sicau. 1B-1	1B	SNP1110	Resistance to FCR	27. 78 ~ 28. 66	Hou et al. (2023)
Qcr. sicau. 1B-2	1B	SNP1607	Resistance to FCR	530. 24 ~ 532. 25	Hou et al. (2023)
Qfcr. sicau. 1B-3	1B	SNP1766	Resistance to FCR	607. 16	Hou et al. (2023)
Qcr-Affx-109205872	1D	Affx-109205872	Resistance to FCR	131. 29	Jin et al (2020)
Qcr-Xgwm95	2A	Xgwm95-Xcfa2043	Resistance to FCR	309. 39	Martin et al (2015)
Qfcr. sicau. 2B-1	2B	SNP5583	Resistance to FCR	794. 74	Hou et al. (2023)
QCr. usq-2B. 2	2B	WFt-5374-wPt-0434	Resistance to FCR	463. 77	Bovill et al. (2010)
Qfcr. sicau. 2B-2	2B	SNP5655	Resistance to FCR	809. 62	Hou et al. (2023)
Qfcr. sicau. 2D-1	2D	SNP5957	Resistance to FCR	90. 58	Hou et al. (2023)
QFCR. heau-2D	2D	Xcfd53	Resistance to FCR	23. 02	Yang et al. (2019)
Qfcr. sicau. 2D-2	2D	SNP6065	Resistance to FCR	577. 59	Hou et al. (2023)
Qfcr. sicau. 3B-2	3B	SNP7561	Resistance to FCR	126. 92	Hou et al. (2023)
Qfcr. sicau. 3B-3	3B	SNP7718	Resistance to FCR	262. 57	Hou et al. (2023)
Qcr-wpt-7569PCR	4B	wpt-7569PCR	Resistance to FCR	453. 78	Martin et al. (2015)
Qfcr. sicau. 4B-1	4B	SNP9933	Resistance to FCR	50. 13	Hou et al. (2023)
Qfcr-wPt-3058	4D	wPt-3058	Resistance to FCR	278. 48	Poole et al. (2012)
Ofcr sicau. 5B-1	5B	SNP11592	Resistance to FCR	76. 60	Hou et al. (2023)
QFCR. heau-6A	6A	Xbarc3	Resistance to FCR	85. 28	Yang et al. (2019)
Ofcr sicau. 7A-1	7A	SNP16572	Resistance to FCR	614. 69	Hou et al. (2023)
Ofcr-sicau. 7B-1	7B	SNP17158	Resistance to FCR	49. 03	Hou et al. (2023)

In recent years, genome-wide association study (GWAS) based on natural populations has been widely used in the detection of resistance loci to FCR (Yang et al., 2025). Through GWAS analysis of a large number of wheat varieties, multiple loci related to FCR resistance have been discovered, and these loci are distributed on different chromosomes of wheat (Wang et al., 2024; Yang et al., 2025). Currently, there are as many as 140 reported resistance loci to FCR in wheat, which are almost distributed on all 21 chromosomes of wheat (Li et al., 2024b). Among them, the resistance loci on chromosomes 1B, 2B, 3B, 4B, 6A, and 6B are relatively abundant, and these chromosomal regions may contain more genes related to FCR resistance. Loci such as Qcr.usq-4B.1, Qcrs.cpi-3B (Qcrs.wsu-3BL), and Qcr.usq-4B.1 have relatively large effects and can be stably detected in different studies, indicating that these loci play an important role in wheat FCR resistance and have high application value (Li et al., 2024a).

However, although many resistance loci have been identified, there are currently few reports of major loci or genes being actually applied in breeding. This is mainly because the genetic mechanism of wheat FCR resistance is relatively complex, involving interactions between multiple genes as well as the interaction between genes and the environment, which makes it difficult to effectively apply these resistance loci to breeding practices. In addition, the fine mapping and cloning of resistance loci still need to be further carried out to further clarify their functions and action mechanisms, providing more accurate theoretical support for disease-resistant breeding.

### 5.3 Discovered Genes and Functional Analysis

In recent years, with the continuous development of molecular biology technology, remarkable progress has been made in the discovery of genes resistant to FCR in wheat. Multiple genes regulating resistance have been identified, which enhance wheat’s resistance to FCR through different mechanisms. The wheat receptor-like protein coding gene TaRLK-6A is one of the important resistance genes. TaRLK-6A interacts with the somatic embryogenesis receptor-like kinase TaSERK1 and positively regulates the expression of defense genes such as TaMPK3, TaERF3, TaDefensin, TaPR1, and TaChitinase, thereby activating the defense response of wheat and improving wheat’s resistance to FCR (Qi et al., 2024). This study reveals the important action mechanism of TaRLK-6A in the process of wheat resistance to FCR, providing an important candidate gene for molecular breeding of wheat resistance to FCR.

The cell wall-associated kinase gene TaWAK-5D600 also plays an important role in wheat FCR resistance (Qi et al., 2023). Studies have shown that they regulate the resistance to wheat sharp eyespot and FCR through similar mechanisms. These genes may be involved in the metabolism and signal transduction processes of the cell wall, and resist pathogen infection by enhancing the strength and stability of the cell wall. TaWAK-5D600 may interact with other components in the cell wall, regulate the synthesis and modification of the cell wall, and thus affect the invasion and colonization of pathogens. In addition, they may also activate the downstream defense signal pathway, induce the expression of defense genes, and enhance the disease resistance of wheat.

The cytosolic acetoacetyl-CoA thiolase II (AACT) gene TaAACT1 is also a gene that positively regulates wheat FCR resistance (Xiong et al., 2023). AACT is a key enzyme in the plant terpenoid synthesis pathway and is involved in the synthesis of various secondary metabolites. The study found that TaAACT1 may affect wheat’s disease resistance by regulating the synthesis of terpenoids. Terpenoids have various biological activities such as antibacterial and antioxidant activities, and can enhance the plant’s resistance to pathogens. TaAACT1 may enhance wheat’s resistance to FCR by promoting the synthesis of terpenoids and increasing the content of defense substances in wheat.

The heterologous expression of the barley transcription factor gene HvWRKY6 and the uridine diphosphate-dependent glucosyltransferase gene HvUGT13248 in wheat can also enhance wheat’s resistance to FCR (Li et al., 2022; Mandalà et al., 2019). HvWRKY6 is a transcription factor that can regulate the expression of downstream defense genes. After expressing HvWRKY6 in wheat, it may activate the defense signal pathway of wheat itself, induce the expression of a series of defense genes, and thus enhance the disease resistance of wheat. HvUGT13248 may participate in the disease resistance process of wheat by catalyzing the glycosylation modification of the substrate and changing the biological activity of the substrate. Glycosylation modification is an important metabolic regulation method in plants, which can affect the activity of plant hormones, signal transduction, and the synthesis and accumulation of secondary metabolites.

The cysteine-rich repeat receptor-like kinase gene TaCRK-7A, the cell wall invertase gene TaCWI-B1, and the Fusarium head blight resistance gene Fhb7 encoding glutathione S-transferase (GST) have also been proven to positively regulate wheat FCR resistance (Wu et al., 2021; Lv et al., 2023). TaCRK-7A may activate the downstream defense response by sensing the invasion signal of pathogens; TaCWI-B1 may resist pathogen infection by regulating the metabolism of the cell wall and enhancing the strength and stability of the cell wall; the glutathione S-transferase encoded by Fhb7 can catalyze the binding reaction of glutathione and electrophilic substances, participate in the detoxification process in plants, and may enhance wheat’s resistance to FCR by detoxifying the toxins produced by fungi.

The high-throughput panorama of transcriptomics, proteomics, phosphoproteomics, and acetylproteomics reveals a new mechanism by which the TaHDA9-TaP5CS1 module enhances wheat’s resistance to FCR through the regulation of proline (Zhang et al., 2025). In tobacco and wheat protoplasts, it has been confirmed that the phosphorylation of TaCAT2-R by TaSnRK1α can enhance the protein stability of the latter, thereby enhancing the ability of TaCAT2-R to scavenge reactive oxygen species in plants, and thus regulating wheat FCR resistance (Yang et al., 2025).

**Figure 5:**
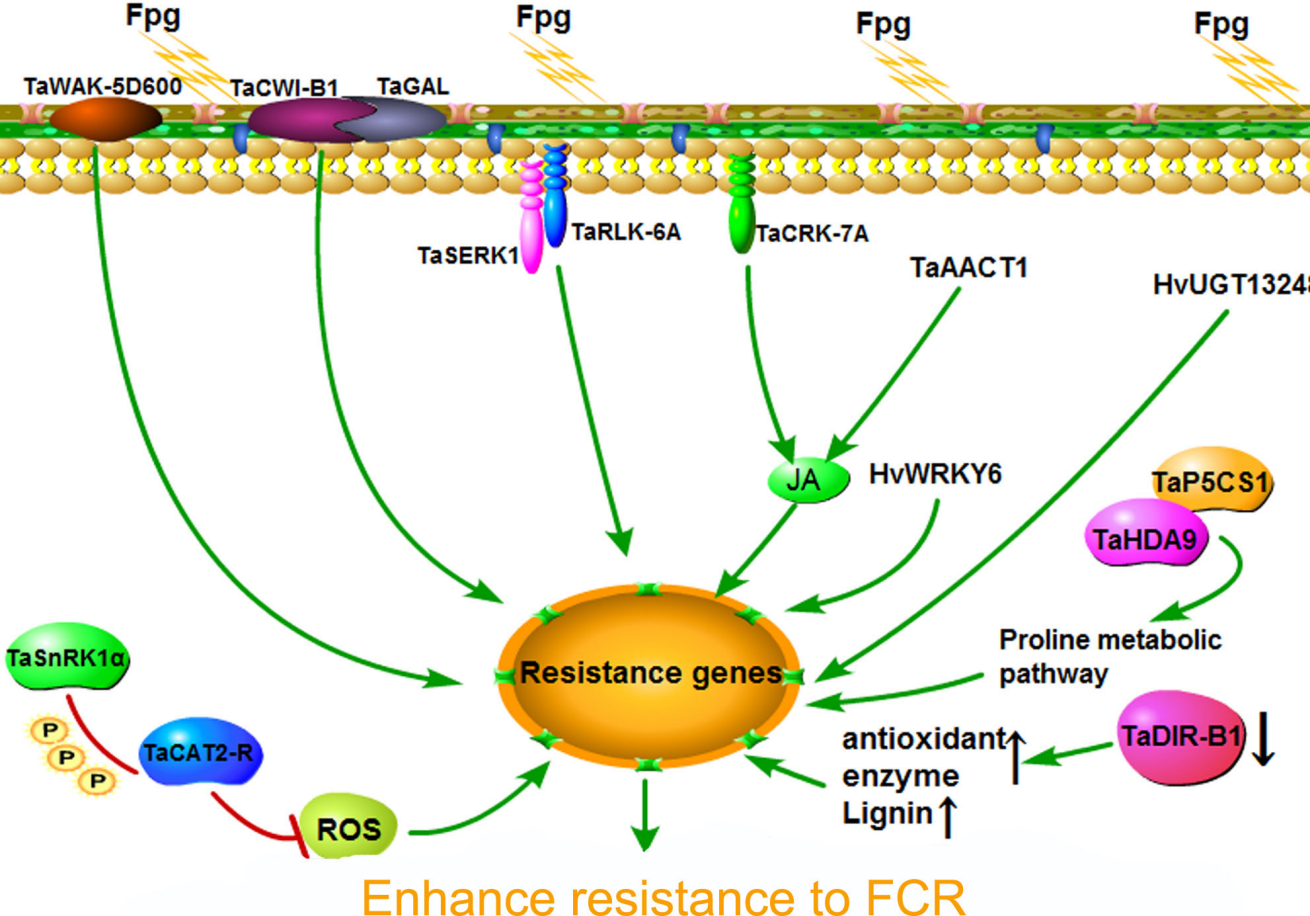
Research progress of genes resistant to fusarium crown rot of wheat.

Compared with genes that positively regulate resistance, there are relatively few reports of genes that negatively regulate wheat FCR resistance. Among them, TaDIR-B1 is a negatively regulating resistance gene that has been more deeply studied at present (Yang et al., 2021). The TaDIR-B1 gene in the highly susceptible material Pingyuan 50 was silenced by VIGS (virus-induced gene silencing) technology. The results showed that the resistance of the silenced plants to wheat FCR was significantly enhanced, and the lignin content and antioxidant enzyme activity in the silenced plants increased significantly. In addition, researchers also screened tetraploid wheat mutants and hexaploid wheat mutants of the TaDIR-B1 gene, and found that the functional deficiency of the TaDIR-B1 gene could significantly enhance the resistance of wheat to FCR, and the lignin content and oxidase activity were significantly enhanced. Comprehensive above research results indicate that the TaDIR-B1 gene may regulate wheat FCR resistance by adjusting the lignin content in the plants (**Figure 5**).

## 6 .Conclusions

Fusarium crown rot (FCR) of wheat poses a serious threat to global wheat production. The discovery of disease-resistant genes and the analysis of their mechanisms play a crucial role in the prevention and control of this disease. Currently, with the aid of advanced molecular biology and genomics technologies, remarkable progress has been made in the discovery of disease-resistant genes, and numerous gene loci related to disease resistance have been identified. Meanwhile, research on disease resistance mechanisms has been continuously deepened. It has been clarified that disease-resistant genes resist pathogen invasion through multiple pathways, such as activating the immune system, regulating the expression of defense genes, enhancing cell wall stability, and mediating reactive oxygen species metabolism, with signal transduction pathways playing an important role in this process. These achievements provide important gene resources for the genetic improvement of wheat, facilitate the breeding of new wheat varieties with high resistance to FCR, and lay a solid foundation for the sustainable prevention and control of the disease. However, there are still some limitations in current research. For example, the transformation efficiency of identified disease-resistant genes in practical breeding applications needs to be improved, and the details of some disease resistance mechanisms remain unclear. In the future, it is necessary to further strengthen interdisciplinary integration, comprehensively use means such as genetics, biochemistry, and molecular biology to deeply analyze the action networks and fine regulatory mechanisms of disease-resistant genes, and accelerate the transformation process of disease-resistant genes from laboratory to field applications. Meanwhile, emerging technologies such as gene editing and big data analysis should be used to discover more excellent disease-resistant genes, broaden the genetic basis of wheat disease resistance, and provide more powerful technical support for ensuring global wheat production safety.

Looking ahead, an efficient screening system for disease-resistant genes based on multi-omics data and a targeted improvement strategy using CRISPR-Cas9 precision editing technology will become core directions for breaking through existing research bottlenecks. For instance, the combined analysis of transcriptomics and metabolomics to analyze the spatiotemporal expression networks of disease-resistant genes, coupled with gene editing technology to optimize disease resistance signal pathways at specific sites, is expected to improve the transformation efficiency of disease-resistant genes while achieving synergistic improvement of multiple resistance traits. Furthermore, the integrated application of artificial intelligence-driven genome-wide association studies (GWAS) and phenomics technologies can accelerate the discovery of minor-effect disease-resistant genes hidden in complex genetic backgrounds, providing a new technical pathway for constructing broad-spectrum and durable disease-resistant breeding systems.

## References

[B1] Beccari G. Prodi A. Pisi A. Nipoti P. Onofri A. Nicholson P.. (2018). Development of three Fusarium crown rot causal agents and systemic translocation of deoxynivalenol following stem base infection of soft wheat. Plant Pathology. 67 , 1055–1065. doi: 10.1111/ppa.12821

[B2] Bovill W. D. Horne M. Herde D. Davis M. Wildermuth G. B. Sutherland M. W. (2010). Pyramiding QTL increases seedling resistance to crown rot (*Fusarium pseudograminearum*) of wheat (*Triticum aestivum*). Theor. Appl. Geneties 121, 127–136. doi: 10.1007/s00122-010-1296-7, PMID: 20198470

[B3] Brennan J. M. Fagan B. Maanen A. V. Cooke B. M. Doohan F. M. (2003). Studies on *in vitro* growth and pathogenicity of european fusarium fungi. Eur. J. Plant Pathol. 109, 577–587. doi: 10.1023/A:1024712415326

[B4] Cheng L. G. (2013). Genetics (Beijing: Science Press).

[B5] Chimthai S. Cheabu S. Aesomnuk W. Ruengphayak S. Arikit S. Vanavichit A.. (2025). Breeding for heat tolerant aromatic rice varieties and identification of novel qtl regions associated with heat tolerance during reproductive phase by qtl-seq. Rice Sci. 32, 67–80. doi: 10.1016/j.rsci.2024.12.002

[B6] Darvasi A. Weinreb A. Minke V. Weller J. I. Soller M. (1993). Detecting marker-QTL linkage and estimating QTL gene effect and map location using a saturated genetic map. Genetics 134, 943–951. doi: 10.1101/gad.7.7b.1459, PMID: 8349116 PMC1205528

[B7] Fan X. Yan Z. Yang M. Waalwijk C. van der Lee T. van Diepeningen A.. (2021). Contamination and translocation of deoxynivalenol and its derivatives associated with fusarium crown rot of wheat in northern China. Plant Dis. 105, 3397–3406. doi: 10.1094/PDIS-03-21-0612-RE, PMID: 33944574

[B8] Glenn A. E. Gold S. E. Bacon C. W. (2002). Fdb1 and Fdb2, *Fusarium verticillioides* loci necessary for detoxification of preformed antimicrobials from corn. Mol. Plant-Microbe Interact. 15, 91–101. doi: 10.1094/MPMI.2002.15.2.91, PMID: 11876429

[B9] Gu Y. Guo H. Li H. Su R. Khan N. U. Li J.. (2024). Qtl mapping by gwas and functional analysis of osbzip72 for cold tolerance at rice seedling stage. Crop J. 12, 1697–1708. doi: 10.1016/j.cj.2024.07.014

[B10] Hou S. Lin Y. Yu S. Yan N. Chen H. Shi H.. (2023). Genome-wide association analysis of Fusarium crown rot resistance in Chinese wheat landraces. Theor. Appl. Genet. 136, 101. doi: 10.1007/s00122-023-04289-y, PMID: 37027037

[B11] Huo Y. Zhang P. Ren L. J. Yao J. B. Ma H. X. (2010). Study on the rapid inoculation and identification method of wheat crown rot at the seedling stage. Acta Agriculturae Jiangxi 8), 4. doi: 10.3969/j.issn.1001-8581.2010.08.029

[B12] Jin J. Duan S. Qi Y. Yan S. Li W. Li B.. (2020). ldentification of anovel genomie region associated with resistanee to Fusa.rium crown rot in wheat. Theor. Appl. Genet. 133, 2063–2073. doi: 10.1007/s00122-020-03577-1, PMID: 32172298

[B13] Kettle A. J. Batley J. Benfield A. H. Manners J. M. Kazan K. Gardiner D. M. (2015). Degradation of the benzoxazolinone class of phytoalexins is important for virulence of fusarium pseudograminearum towards wheat. Mol. Plant Pathol. 16, 946–962. doi: 10.1111/mpp.12250, PMID: 25727347 PMC6638480

[B14] Li J. Zhai S. Xu X. Su Y. Yu J. Gao Y. (2024b). Dissecting the genetic basis of Fusarium crown rot resistance in wheat by genome wide association study. TAG. Theoretical and applied genetics. Theoretische und angewandte Genetik. 137, 43. doi: 10.1007/s00122-024-04553-9, PMID: 38321245

[B15] Li M. Zhao S. Yang J. Ren Y. Su J. Zhao J. (2022). Exogenous expression of barley HvWRKY6 in wheat improves broad-spectrum resistance to leaf rust, Fusarium crown rot, and sharp eyespot. Int. J. Biol. macromolecules. 218, 1002–1012. doi: 10.1016/j.ijbiomac.2022.07.138, PMID: 35872316

[B16] Li Q. Y. Hao X. P. Jiang Y. M. Guo Z. F. Niu J. S. Yin G. H. (2024a). Research progress on resistance loci of wheat crown rot. J. Henan Agric. University. 58, 539–551. doi: 10.16445/j.cnki.1000-2340.20240320.001

[B17] Li X. Liu C. Chakraborty S. Manners J. M. Kazan K. (2008). A simple method for the assessment of crown rot disease severity in wheat seedlings inoculated with *fusarium pseudograminearum* . J. Phytopathol. 11/12), 156. doi: 10.1111/j.1439-0434.2008.01425.x

[B18] Lin Y. Chen H. Yan N. Li C. Hou S. Mou Y.. (2023). Identification and genome-wide association analysis for fusarium crown rot resistance in wheat. Plant Dis. 107, 1151–1158. doi: 10.1094/PDIS-08-22-1861-RE, PMID: 36306443

[B19] Lv G. Zhang Y. Ma L. Yan X. Yuan M. Chen J. (2023). A cell wall invertase modulates resistance to fusarium crown rot and sharp eyespot in common wheat. J. Integr. Plant Biol. 65, 1814–1825. doi: 10.1111/jipb.13478, PMID: 36912577

[B20] Mandalà G. Tundo S. Francesconi S. Gevi F. Zolla L. Ceoloni C. (2019). Deoxynivalenol detoxification in transgenic wheat confers resistance to fusarium head blight and crown rot diseases. Mol. Plant-Microbe interactions. 32, 583–592. doi: 10.1094/MPMI-06-18-0155-R, PMID: 30422742

[B21] Martin A. Bovill W. D. Percy C. D. Herde D. Fletcher S. Kelly A.. (2015). Mar-kers for seedling and adult plant crown mt resistance infour partially resistant bread wheat sources. Theoretical Appl. Genet. 128, 377–385. doi: 10.1007/s00122-014-2437-1, PMID: 25471673

[B22] McKnight T. Hart J. (1966). Some field observations on crown rot disease of wheat caused by *Fusarium graminearum* . Queensland J. Agric. Anim. Sci. 23, 373–378.

[B23] Mitter V. Francl L. J. Ali S. Simpfendorfer S. Chakraborty S. (2006). Ascosporic and conidial inoculum of gibberella zeae play different roles in fusarium head blight and crown rot of wheat in Australia and the USA. Australasian Plant Pathology 35(4), 441–452. doi: 10.1071/AP06046

[B24] Monds R. D. Cromey M. G. Lauren D. R. Menna M. D. Marshall J. (2005). ). Fusarium graminearum, f. cortaderiae and f. pseudograminearum in New Zealand: molecular phylogenetic analysis, mycotoxin chemotypes and co-existence of species. Mycological Res. 109, 410–420. doi: 10.1017/S0953756204002217, PMID: 15912928

[B25] Morris G. P. Ramu P. Deshpande S. P. Hash C. T. Shah T. Upadhyaya H. D. (2013). Population genomic and genome-wide association studies of agroclimatic traits in sorghum. Proc. Natl. Acad. Sci. United States America. 110, 453–458. doi: 10.1073/pnas.1215985110, PMID: 23267105 PMC3545811

[B26] Poole G. J. Smiley R. W. Paultz T. C. Walker C. A. Carter A. H. See D. R.. (2012). Identification of quantitative trait loci (QTL) for resistance to Fusarium crown rot (*Fusarium pseudograminearum*) in multiple assay environments in the Pacific Northwestern US. Theor. Appl. Genet. 125, 91–107. doi: 10.1007/s00122-012-1818-6, PMID: 22366812 PMC3351592

[B27] Qi H. Zhu X. Shen W. Yang X. Zhang C. Li G. (2024). TaRLK-6A promotes Fusarium crown rot resistance in wheat. J. Integr. Plant Biol. 66, 12–16. doi: 10.1111/jipb.13596, PMID: 38103031

[B28] Qi H. Zhu X. Shen W. Zhang Z. (2023). A novel wall-associated kinase TaWAK-5D600 positively participates in defense against sharp eyespot and Fusarium Crown Rot in wheat. Int. J. Mol. Sci. 24, 5060. doi: 10.3390/ijms24055060, PMID: 36902488 PMC10003040

[B29] Shi M. L. Yuan Z. Ma J. P. Xu Q. Zhang M. H. (2024). Research progress on the occurrence and control of wheat crown rot caused by fusarium pseudograminearum in Henan province. Bull. Agric. Sci. Technology. 11), 129–132.

[B30] Smiley R. W. Machado S. (2020). Fusarium crown rot of winter wheat influenced by resource competition near a tree windbreak. Plant Dis. 104, 348–357. doi: 10.1094/pdis-01-19-0213-re, PMID: 31841102

[B31] Smiley R. W. Gourlie J. A. Easley S. A. Patterson L. M. (2005). Pathogenicity of fungi associated with the wheat crown rot complex in oregon and washington. Plant Dis. 89, 949–957. doi: 10.1094/PD-89-0949, PMID: 30786628

[B32] Sun H. Cai S. Deng Y. Cao S. Yang X. Lu Y.. (2024). Efficacy of cyclobutrifluram in controlling Fusarium crown rot of wheat and resistance risk of three Fusarium species to cyclobutrifluram. Pesticide Biochem. Physiol. 198, 105723. doi: 10.1016/j.pestbp.2023.105723, PMID: 38225078

[B33] Tian Z. W. Zhang Z. Wang G. J. He Z. J. Wang X. E. Bai L. H. (2015). Preliminary study on wheat crown rot. Shaanxi J. Agric. Sci. 61, 18–19. doi: 10.3969/j.issn.0488-5368.2015.03.008

[B34] Wang C. Sun M. Zhang P. Ren X. Zhao S. Li M.. (2024). Genome-wide association studies on chinese wheat cultivars reveal a novel fusarium crown rot resistance quantitative trait locus on chromosome 3bl. Plants 2223-7747). 13, 856. doi: 10.3390/plants13060856, PMID: 38592894 PMC10974656

[B35] Wu T. Guo F. Xu G. Yu J. Zhang L. Wei X.. (2021). The receptor-like kinase taCRK-7A inhibits fusarium pseudograminearum growth and mediates resistance to fusarium crown rot in wheat. Biology. 10, 1122. doi: 10.3390/biology10111122, PMID: 34827115 PMC8614996

[B36] Wu B. Guo X. Zhang M. Jiang S. S. Xin Z. M. Wang S. J.. (2018). Identification of the pathogenic bacteria of wheat stem base rot in southwestern Shandong Province and analysis of their pathogenicity. J. Triticeae Crops 38, 8. doi: 10.7606/j.issn.1009-1041.2018.03.14

[B37] Xiong F. Zhu X. Luo C. Liu Z. Zhang Z. (2023). The Cytosolic Acetoacetyl-CoA Thiolase TaAACT1 Is Required for Defense against Fusarium pseudograminearum in Wheat. Int. J. Mol. Sci. 24, 6165. doi: 10.3390/ijms24076165, PMID: 37047146 PMC10094598

[B38] Xu L. Meng X. Guo L. Li J. D. Zhao S. H. Wang X. H.. (2025). Field control effect test of different biological agents in the control of wheat stem base rot. J. Northeast Agric. Sci. 50, 10–15.

[B39] Xu F. Song Y. L. Zhou Y. L. Zhang H. Wang J. M. Li Y. H.. (2016). Occurrence, damage situation and characteristics of wheat stem base rot in Henan Province from 2013 to 2016. Plant Prot. 42, 7. doi: 10.3969/j.issn.05291542.2016.06.023

[B40] Xu X. Yu T. F. Wei J. T. Ma X. F. Liu Y. W. Zhang J. P.. (2024). TaWRKY24 integrates the tryptophan metabolism pathways to participate in defense against Fusarium crown rot in wheat. Plant journal: Cell Mol. Biol. 120, 1764–1785. doi: 10.1111/tpj.17079, PMID: 39499237

[B41] Yang X. Pan Y. Singh P. K. He X. Ren Y. Zhao L.. (2019). Investigationand genome-wide association study for Fusarium crownrot resistance in Chinese common wheat. BMC Plant Biol. 19 (1), 153. doi: 10.1186/s12870-019-1758-2, PMID: 31014249 PMC6480828

[B42] Yang X. Zhang L. Wei J. Liu L. Liu D. Yan X.. (2025). A TaSnRK1α-TaCAT2 model mediates resistance to Fusarium crown rot by scavenging ROS in common wheat. Nat. Commun. 16, 2549. doi: 10.1038/s41467-025-57936-x, PMID: 40089587 PMC11910652

[B43] Yang X. Zhong S. Zhang Q. Ren Y. Sun C. Chen F. (2021). A loss-of-function of the dirigent gene TaDIR-B1 improves resistance to Fusarium crown rot in wheat. Plant Biotechnol. J. 19, 866–868. doi: 10.1111/pbi.13554, PMID: 33567136 PMC8131038

[B44] Ye S. Y. (2009). Construction of the genetic map of torreya grandis and the mapping of QTLs related to seedling growth (Zhejiang, China: Zhejiang Forestry College).

[B45] Yu S. F. (2021). Identification of resistance to wheat crown rot in local wheat varieties and genome-wide association analysis (Sichuan, China: Sichuan Agricultural University). doi: 10.27345/d.cnki.gsnyu.2021.000413

[B46] Zhang N. Tang L. Li S. Liu L. Gao M. Wang S.. (2025). Integration of multi-omics data accelerates molecular analysis of common wheat traits. Nat. Commun. 16, 2200. doi: 10.1038/s41467-025-57550-x, PMID: 40038279 PMC11880479

[B47] Zhang L. L. Yan X. G. Yuan M. J. Jian J. T. Wei J. J. Li J. Q.. (2024). Identification and mapping analysis of resistance to sharp eyespot of wheat germplasm resources. J. Plant Genet. Resour. 25, 184–192. doi: 10.13430/j.cnki.jpgr.20230718001

[B48] Zhang J. Zhang D. Fan Y. Li C. Xu P. Li W.. (2021). The identification of grain size genes by RapMap reveals directional selection during rice domestication. Nat. Commun. 12, 5673. doi: 10.1038/s41467-021-25961-1, PMID: 34584089 PMC8478914

[B49] Zhao M. Wang W. Chen W. Ma C. Zhang F. Jiang K. (2019). Genome survey, high-resolution genetic linkage map construction, growth-related quantitative trait locus (QTL) identification and gene location in Scylla paramamosain. Sci. Rep. 9, 2910. doi: 10.1038/s41598-019-39070-z, PMID: 30814536 PMC6393678

